# Comparing chemotherapy-induced nausea and vomiting between young adult vs non-young adult cancer patients

**DOI:** 10.1093/jncics/pkag082

**Published:** 2026-08-18

**Authors:** Laura B Oswald, Aasha I Hoogland, Taylor L Welniak, Oanh L Nguyen, Yvelise Rodriguez, Xiaoyin Li, Samantha Reese, Paige W Lake, Brian D Gonzalez, Martine Extermann, Jonathan Metts, Matthew A Murphy, Brent J Small, Donna L Berry, Daneng Li, Kristen M Carpenter, Stacy M Fischer, Anita Y Kinney, Heather S L Jim

**Affiliations:** Department of Health Outcomes and Behavior, Moffitt Cancer Center, Tampa, FL, United States; Department of Health Outcomes and Behavior, Moffitt Cancer Center, Tampa, FL, United States; Department of Health Outcomes and Behavior, Moffitt Cancer Center, Tampa, FL, United States; Department of Health Outcomes and Behavior, Moffitt Cancer Center, Tampa, FL, United States; Department of Health Outcomes and Behavior, Moffitt Cancer Center, Tampa, FL, United States; Department of Health Outcomes and Behavior, Moffitt Cancer Center, Tampa, FL, United States; Department of Health Outcomes and Behavior, Moffitt Cancer Center, Tampa, FL, United States; Department of Health Outcomes and Behavior, Moffitt Cancer Center, Tampa, FL, United States; Department of Health Outcomes and Behavior, Moffitt Cancer Center, Tampa, FL, United States; Senior Adult Oncology Program, Moffitt Cancer Center, Tampa, FL, United States; Sarcoma Department, Moffitt Cancer Center, Tampa, FL, United States; Cancer and Blood Disorders Institute, Johns Hopkins All Children’s Hospital, St. Petersburg, FL, United States; Department of Supportive Care Medicine, Moffitt Cancer Center, Tampa, FL, United States; School of Nursing, University of North Carolina at Chapel Hill, Chapel Hill, NC, United States; School of Nursing, University of Washington, Seattle, WA, United States; Department of Medical Oncology and Therapeutics Research, City of Hope Comprehensive Cancer Center, Duarte, CA, United States; Department of Psychiatry and Behavioral Health, The Ohio State University, Columbus, OH, United States; Department of General Internal Medicine, University of Colorado Denver, Aurora, CO, United States; Department of Biostatistics and Epidemiology, School of Public Health, Rutgers University, Piscataway, NJ, United States; Department of Health Outcomes and Behavior, Moffitt Cancer Center, Tampa, FL, United States

## Abstract

Chemotherapy-induced nausea and vomiting are among the most distressing side effects of cancer therapy. Younger age is a risk factor for chemotherapy-induced nausea and vomiting, and cancer incidence is rising among young adults ages 18-39 years. However, the comparative burden of chemotherapy-induced nausea and vomiting in young adults vs non-young adults remains insufficiently understood. This study compared acute and delayed chemotherapy-induced nausea and vomiting between young adults and non-young adults (ie, ages 40 years and older) after the first infusion of moderately or highly emetogenic chemotherapy. Of 1609 participants, 159 (10%) were young adults. Rates of guideline-consistent antiemetic prophylactic care were similar between groups (65% young adults, 71% non-young adults; *P *= .11). Despite this, acute nausea was more prevalent (69% vs 40%; *P *< .001) and severe (mean [SD] = 3.9 [2.6] vs mean = 3.0 [2.3] on 0-10 scale; *P *< .001) among young adults vs non-young adults. Similarly, delayed nausea was more prevalent (85% vs 69%; *P *< .001) and severe (mean = 4.5 [2.4] vs mean = 3.7 [2.5]; *P *< .001) among young adults. Findings may inform clinical approaches to managing chemotherapy-induced nausea and vomiting among young adults.

Chemotherapy-induced nausea and vomiting are some of the most feared side effects of cancer treatment^1-3^ and substantially impact patients’ quality of life.^4-6^ There have been substantial advancements in prophylactic antiemetics with increased use of 5-HT3 receptor antagonists, NK1 receptor antagonists, and olanzapine-based regimens.^7^^,^^8^ However, existing regimens primarily control vomiting, not nausea. Moreover, the application of guideline-consistent antiemetic care is inconsistent in practice, and chemotherapy-induced nausea and vomiting persists for a substantial proportion of patients even when guideline-consistent antiemetics are administered.^9^

One of the strongest predictors of chemotherapy-induced nausea and vomiting is age, with younger patients more likely to experience chemotherapy-induced nausea and vomiting than older patients receiving the same treatment.^10^^,^^11^ Given the rising cancer incidence among young adults ages 18-39 years,^12^ this signals a potential need for more aggressive antiemetic care among young adults. However, most prior research examining age as a predictor of chemotherapy-induced nausea and vomiting aggregated younger patients into broad categories (eg, ages younger than 50 years or younger than 65 years)^10^ rather than focusing specifically on young adults, leaving young adults underrepresented and poorly characterized. Consequently, the comparative burden of chemotherapy-induced nausea and vomiting in young adults vs non-young adults (ie, ages 40 years and older) remains insufficiently understood. To address this gap, we compared the incidence and severity of acute and delayed chemotherapy-induced nausea and vomiting between young adult and non-young adult patients after the first infusion of moderately or highly emetogenic chemotherapy, compared chemotherapy-induced nausea and vomiting management strategies between young adults and non-young adults, and explored risk factors for nausea among young adults.

This was a secondary analysis from a larger observational study.^13-16^ The protocol was approved by Western Institutional Review Board (Identifier: 20180593). Participants were recruited between March 2019 and March 2023 at Moffitt Cancer Center (Tampa, FL, USA), City of Hope Comprehensive Cancer Center (Duarte, CA, USA), The Ohio State University (Columbus, OH, USA), Rutgers University (Piscataway, NJ, USA), and University of Colorado Denver (Aurora, CO, USA). Eligible patients were (1) aged 18 years and older, (2) able to speak and read English or Spanish, (3) diagnosed with a solid tumor, (4) chemotherapy-naïve, (5) scheduled for moderately or highly emetogenic intravenous standard-dose chemotherapy (according to most recent National Comprehensive Cancer Network [NCCN] antiemesis guidelines), and (6) able to provide informed consent. Exclusion criteria included a diagnosis of inflammatory bowel disease, a nasogastric tube, or total parenteral nutrition. Participants completed chemotherapy-induced nausea and vomiting assessments on paper or electronically via an online platform (DatStat).

At baseline, participants reported their demographics and completed the patient-reported Charlson Comorbidity Index.^17^^,^^18^ Clinical information was abstracted from medical records. Acute and delayed chemotherapy-induced nausea and vomiting were assessed using the Multinational Association for Supportive Care and Cancer Antiemesis Tool.^19^ Acute chemotherapy-induced nausea and vomiting was assessed approximately 24 hours after participants’ first chemotherapy infusion. Delayed chemotherapy-induced nausea and vomiting was assessed approximately 5 days later. At each timepoint, participants reported the incidence (yes, no) of nausea and, if endorsed, its severity from 0 (none) to 10 (as much as possible). Participants also reported the incidence (yes, no) of vomiting and, if endorsed, its frequency (eg, how many times they vomited). At both timepoints, participants endorsing chemotherapy-induced nausea and vomiting selected how they managed their symptoms from a list of 12 symptom management strategies, including an option to write in strategies not listed.

The χ^2^ and Wilcoxon rank sum tests were used to compare the incidence (with 95% Wilson confidence interval [CI]) and severity or frequency of acute and delayed chemotherapy-induced nausea and vomiting between young adults and non-young adults. Odds ratios and Wilcoxon effect sizes (Z/√N) were used to examine the magnitude of group differences (Wilcoxon *r* = 0.10 small, *r* = 0.30 medium, *r* = 0.50 large). The χ^2^, Fisher exact tests, independent sample *t* tests, and Wilcoxon rank sum tests were used to compare rates of chemotherapy-induced nausea and vomiting symptom management strategies between young adults and non-young adults. Univariate logistic regression was used to explore relationships between patient characteristics and acute and delayed nausea rated moderate or worse (ie, ≥4 of 10) among young adults.

Of 1609 participants, 10% (n = 159) were young adults (Table S1). On average, young adults were aged 34 years (SD = 3.99 years, range = 22-39 years) and non-young adults (n = 1450) were aged 61 years [SD = 10.79 years; range = 40-90 years). A larger proportion of young adults were female (83% vs 71%; *P *= .002), Hispanic (16% vs 10%; *P* = .03), not married (42% vs 32%; *P* = .01), college graduates (62% vs 49%; *P* = .001), with early stage disease (84% vs 76%; *P* = .02). Young adults also reported fewer comorbidities (*P* < .001). There were group differences in disease site, with young adults more likely to be diagnosed with breast cancer (63% vs 37%; *P* < .001). Similar proportions of young adults and non-young adults were undergoing moderately (70% vs 65%) and highly emetogenic chemotherapy (30% vs 35%; *P* = .22) and received guideline-consistent antiemetic care (65% vs 71%; *P* = .10), according to most recent NCCN antiemesis guidelines.

Overall, 40% of participants endorsed acute chemotherapy-induced nausea and vomiting and 66% endorsed delayed chemotherapy-induced nausea and vomiting. Table 1 shows the incidences and severities or frequencies by age group. For acute chemotherapy-induced nausea and vomiting, a larger proportion of young adults reported acute nausea (69% [95% CI = 61% to 76%] vs 40% [95% CI = 37% to 42%]; *P* < .001), and acute nausea was more severe among young adults than non-young adults (mean [SD] = 3.9 [2.6] vs 3.0 [2.3]; *P* < .001). A larger proportion of young adults reported acute vomiting (7% [95% CI = 4% to 12%] vs 4% [95% CI = 3% to 5%]; *P* = .03), although acute vomiting was rare overall, and average frequency of acute vomiting was similar between young adults and non-young adults (mean = 3.0 [2.3] vs 2.1 [1.9]; *P* = .18). For delayed chemotherapy-induced nausea and vomiting, a larger proportion of young adults reported delayed nausea (85% [95% CI = 79% to 90%] vs 69% [95% CI = 66% to 71%]; *P* < .001), and delayed nausea was more severe among young adults than non-young adults (mean = 4.5 [2.4] vs 3.7 [2.5]; *P* < .001). There were no group differences in the incidence (18% [95% CI = 13% to 25%] vs 13% [95% CI = 12% to 15%]; *P* = .11) or frequency of delayed vomiting (mean = 4.0 [4.4] vs 3.6 [5.0]; *P* = .71).

**Table 1.** Incidence and severity or frequency of acute and delayed nausea and vomiting among young adults ages 18-39 years and non-young adults ages 40 years and older.^a^

	Young adults (n = 159)		Non-young adults (n = 1450)		*P*	Effect size
Acute nausea						
Incidence	105 (69%)	95% CI = 61% to 76%	542 (40%)	95% CI = 37% to 42%	<.001	OR = 3.27 [95% CI = 2.32 to 4.59]
Severity, mean (SD)	3.9 (2.6)		3.0 (2.3)		<.001	Wilcoxon *r* = 0.14
Acute vomiting						
Incidence	11 (7%)	95% CI = 4% to 12%	49 (4%)	95% CI = 3% to 5%	.03	OR = 2.13 [95% CI = 1.06 to 4.03]
Frequency, mean (SD)	3.0 (2.3)		2.1 (1.9)		.18	Wilcoxon *r* = 0.17
Delayed nausea						
Incidence	127 (85%)	95% CI = 79% to 90%	928 (69%)	95% CI = 66% to 71%	<.001	OR = 3.28 [95% CI = 2.09 to 5.29]
Severity, mean (SD)	4.5 (2.4)		3.7 (2.5)		<.001	Wilcoxon *r* = 0.13
Delayed vomiting						
Incidence	27 (18%)	95% CI = 13% to 25%	182 (13%)	95% CI = 12% to 15%	.11	OR = 1.46 [95% CI = 0.95 to 2.22]
Frequency, mean (SD)	4.0 (4.4)		3.6 (5.0)		.71	Wilcoxon *r* = 0.02

Abbreviation: OR = odds ratio.

a Acute nausea and vomiting were assessed approximately 24 hours after participants’ first chemotherapy infusion. Delayed nausea and vomiting were assessed 5 days after participants’ first chemotherapy infusion.

Table 2 shows the chemotherapy-induced nausea and vomiting management strategies reported by participants by age group. For acute chemotherapy-induced nausea and vomiting, management strategies were similar among young adults and non-young adults (all *P* > .05). The most frequently endorsed acute chemotherapy-induced nausea and vomiting management strategies across young adults and non-young adults were taking prescription antinausea medication (64%) and eating less than usual (27%). Only 15% of participants did nothing to manage their acute chemotherapy-induced nausea and vomiting. For delayed chemotherapy-induced nausea and vomiting, several differences emerged between age groups. A larger proportion of young adults reported taking prescription antinausea medication (84% vs 73%; *P* = .009); eating different foods than usual (26% vs 16%; *P* = .004); drinking liquids but not eating (19% vs 13%; *P* = .050); taking vitamins, herbal supplements, or other remedies (17% vs 7%; *P* < .001); and using marijuana (13% vs 6%; *P* = .003). A smaller proportion of young adults reported doing nothing (3% vs 10%; *P* = .01). Among young adults, no patient or clinical characteristics were associated with reporting at least moderate acute or delayed nausea (all *P*  > .05; Table S2).

**Table 2.** Symptom management strategies reported by young adults ages 18-39 years and non-young adults ages 40 years and older who reported acute and/or delayed chemotherapy-induced nausea and vomiting.^a^

	Acute chemotherapy-induced nausea and vomiting^b^			Delayed chemotherapy-induced nausea and vomiting^b^		
	Young adults, No. (%) (n = 105)	Non-young adults, No. (%) (n = 543)	*P*	Young adults, No. (%) (n = 127)	Non-young adults, No. (%) (n = 930)	*P*
Ate different foods than usual	9 (9%)	40 (7%)	.67	33 (26%)	146 (16%)	.004
Ate less than usual	34 (32%)	144 (27%)	.22	50 (39%)	321 (35%)	.28
Called the doctor	5 (5%)	12 (2%)	.08	10 (8%)	47 (5%)	.19
Did not eat or drink anything	1 (1%)	14 (3%)	.20	8 (6%)	53 (6%)	.15
Drank liquids but did not eat	15 (14%)	76 (14%)	.94	24 (19%)	117 (13%)	.050
Took prescription antinausea medication	74 (70%)	343 (63%)	.15	107 (84%)	683 (73%)	.009
Took nonprescription antinausea medication	1 (1%)	14 (3%)	.20	6 (5%)	43 (5%)	.18
Took vitamins, herbal supplements, or other remedy (eg, ginger tea)	6 (6%)	26 (5%)	.17	22 (17%)	67 (7%)	<.001
Used acupuncture, acupressure, hypnosis, or relaxation techniques	2 (2%)	12 (2%)	.29	4 (3%)	24 (3%)	.20
Used marijuana	6 (6%)	34 (6%)	.18	16 (13%)	53 (6%)	.003
Went to the hospital or urgent care clinic	1 (1%)	8 (1%)	.36	3 (2%)	25 (3%)	.23
If yes, received IV fluids	1 (100%)	5 (63%)	.67	3 (100%)	20 (80%)	.54
Nothing	11 (10%)	85 (16%)	.17	4 (3%)	97 (10%)	.01
Other	10 (10%)	58 (11%)	.69	14 (11%)	77 (8%)	.31

a Categories are not mutually exclusive, and participants could choose as many symptom management strategies as applicable.

b For acute CINV, 542 non-YAs reported acute nausea, and 1 reported acute vomiting without acute nausea, yielding a sample size of 543 non-YAs. For delayed CINV, 928 non-YAs reported delayed nausea, and 5 reported delayed vomiting without delayed nausea, yielding a sample size of 933 non-YAs. Of these, 930 provided data on their delayed CINV symptom management. All YAs who reported acute or delayed vomiting also reported concurrent nausea.

In sum, up to 85% of young adults ages 18-39 years experienced acute and/or delayed nausea after their first infusion of moderately or highly emetogenic chemotherapy, underscoring the pervasiveness of this highly distressing adverse event among young adults. Both acute and delayed nausea were more prevalent and more severe among young adults compared with non-young adults aged 40 years and older. This was one of the largest studies to directly compare the incidence and severity of acute and delayed chemotherapy-induced nausea and vomiting between young adults and non-young adults, offering key insights into the comparative burden of chemotherapy-induced nausea and vomiting between age groups.

High rates of nausea among young adults are notable because 65% of young adults received guideline-consistent antiemetic care, more than in previous reports (eg, 22%-57%).^20-22^ Although encouraging, the high prevalence of nausea among young adults signals an urgent need to better understand the etiology of chemotherapy-induced nausea and vomiting in this population to allow for better symptom control through causal mechanisms and risk factors. Existing risk prediction tools for chemotherapy-induced nausea and vomiting have only moderate predictive accuracy,^23-25^ and we found no relationships between patient and clinical characteristics and reports of at least moderate nausea among young adults. Notably, the relatively small subsample of young adults within the larger study cohort may have limited our statistical power to identify nausea risk factors. Future research should continue to explore these and other potential mechanisms of chemotherapy-induced nausea and vomiting to elucidate novel pathways for better chemotherapy-induced nausea and vomiting prevention and control, such as multi-omics risk factors arising from interactions between host (patient)-related and gut microbial factors.^26^

Although there were no differences by age group with regard to how patients reported managing acute chemotherapy-induced nausea and vomiting, differences emerged for delayed chemotherapy-induced nausea and vomiting. Young adults were more likely to use a variety of management strategies, including marijuana, and were less likely to do nothing. This may indicate an eagerness among young adults for action-focused symptom management strategies.^27^ Future research should investigate the role of behavioral symptom management interventions as a complement to antiemetic regimens to improve chemotherapy-induced nausea and vomiting prevention and control. Notably, this study did not collect data regarding the nature of marijuana use (eg, prescribed vs self-directed) or its perceived efficacy. These findings should not be interpreted as evidence supporting or endorsing marijuana use for chemotherapy-induced nausea and vomiting management.

This study expands prior work that has identified younger age as a risk factor for chemotherapy-induced nausea and vomiting by directly comparing the incidence and severity of acute and delayed chemotherapy-induced nausea and vomiting between young adult and non-young adult patients undergoing moderately or highly emetogenic chemotherapy. As noted, young adults represented a relatively small subsample of the larger cohort. Consequently, adjusted analyses were not feasible, and age-related differences may partially reflect baseline group differences. Additional limitations include broad categories encompassing moderately and highly emetogenic chemotherapy regimens and a predominance of female, non-Hispanic, and White participants. Future studies with larger and more diverse samples of young adults are needed.

## Supplementary Material

pkag082_Supplementary_Data

## Data Availability

The de-identified data that support the findings of this study, including patient-reported outcomes, clinical data, statistical code, and data dictionaries, are available from the principal investigator (Dr Heather Jim) upon reasonable request. Requests must include a written analysis plan describing the proposed research objectives and analytic approach. Requests will be reviewed by the study investigators for scientific merit, feasibility, and consistency with participant consent and applicable regulations. Data access is contingent on the execution of appropriate data-sharing agreements to protect participant confidentiality.
