# Supplementary material for: Comparing chemotherapy-induced nausea and vomiting between young adult vs non-young adult cancer patients
Source: JNCI Cancer Spectr. 2026 Aug 18;10(5):pkag082. doi: 10.1093/jncics/pkag082 (PMC13544965; doi:10.1093/jncics/pkag082)
Supplement: Supplementary file 1 [file pkag082_supplementary_data.docx]

**Table S1.** Characteristics of young adults (YA, ages 18-39) and non-YAs (ages ≥40) receiving moderately or highly emetogenic chemotherapy (N=1,609).

|  | YAs  (n=159) | Non-YAs (n=1,450) | p-value |
| --- | --- | --- | --- |
| Age; mean (SD), range | 34.08 (3.99), 22-39 | 60.81 (10.79), 40-90 | <0.001 |
| Gender; n (%) |  |  | 0.002 |
| Female | 132 (83) | 1,034 (71) |  |
| Male | 27 (17) | 416 (29) |  |
| Race; n (%) |  |  | 0.21 |
| Non-White | 27 (17) | 194 (13) |  |
| White | 132 (83) | 1,256 (87) |  |
| Ethnicity; n (%) |  |  | 0.03 |
| Hispanic | 25 (16) | 148 (10) |  |
| Non-Hispanic | 134 (84) | 1,302 (90) |  |
| Marital status; n (%) |  |  | 0.01 |
| Married | 92 (58) | 979 (68) |  |
| Not married | 67 (42) | 462 (32) |  |
| Education; n (%) |  |  | 0.001 |
| College graduate or above | 99 (62) | 699 (49) |  |
| Not a college graduate | 60 (38) | 731 (51) |  |
| Disease site; n (%) |  |  | <0.001 |
| Breast | 97 (61) | 522 (36) |  |
| Gastrointestinal | 18 (11) | 272 (19) |  |
| Gynecologic | 22 (14) | 278 (19) |  |
| Head and neck | 5 (3) | 145 (10) |  |
| Non-small cell lung cancer | 1 (1) | 70 (5) |  |
| Pancreas | 1 (1) | 92 (6) |  |
| Other | 15 (9) | 70 (5) |  |
| Disease stage; n (%) |  |  | 0.02 |
| Early (1, 2, 3, or limited) | 134 (84) | 1,100 (76) |  |
| Advanced (4 or extensive) | 25 (16) | 344 (24) |  |
| Patient-reported comorbidity index; mean, SD | 2.21 (0.54) | 2.64 (1.16) | <0.001 |
| Recruitment site; n (%) |  |  | 0.15 |
| Moffitt Cancer Center | 30 (19) | 401 (28) |  |
| City of Hope | 24 (15) | 219 (15) |  |
| Ohio State University | 53 (33) | 393 (27) |  |
| Rutgers University | 21 (13) | 160 (11) |  |
| University of Colorado | 31 (20) | 277 (19) |  |
| Treatment emetogenicity; n (%) |  |  | 0.22 |
| Moderate | 111 (70) | 941 (65) |  |
| High | 48 (30) | 508 (35) |  |
| Guideline-consistent antiemetic regimen at first infusion; n (%) |  |  | 0.10 |
| Yes | 103 (65) | 1,028 (71) |  |
| No | 56 (35) | 420 (29) |  |
| Change in antiemetic regimen from first to second infusion; n (%) |  |  | 0.08 |
| Yes | 41 (26) | 281 (20) |  |
| No | 116 (74) | 1,117 (80) |  |

**Table S2.** Odds ratios and 95% confidence intervals for univariate logistic regression analyses examining relationships between patient characteristics and reports of at least moderate acute or delayed nausea (i.e., ≥4 out of 10) among young adults ages 18-39. Significance was indicated by exclusion of the value 1 in the 95% confidence interval. No patient characteristics were significantly associated with acute or delayed nausea rated at least moderate in severity.

|  | Acute nausea | Delayed nausea |
| --- | --- | --- |
| Female (vs. male) | 1.98 (0.69-5.63) | 0.92 (0.39-2.18) |
| White (vs. non-White race) | 0.71 (0.26-1.92) | 1.47 (0.61-3.56) |
| Hispanic (vs. non-Hispanic) | 0.94 (0.38-2.37) | 0.55 (0.23-1.36) |
| Married (vs. not married) | 1.51 (0.74-3.09) | 1.14 (0.60-2.19) |
| College graduate or above (vs. not college graduate) | 1.74 (0.82-3.69) | 0.64 (0.33-1.26) |
| Disease site (vs. breast cancer) |  |  |
| Gynecologic | 1.02 (0.37-2.77) | 1.52 (0.58-3.95) |
| Gastrointestinal | 0.29 (0.06-1.36) | 1.14 (0.39-3.30) |
| Other | 1.02 (0.37-2.77) | 1.71 (0.64-4.57) |
| Advanced disease (vs. early stage) | 0.58 (0.20-1.66) | 0.98 (0.40-2.43) |
| Patient-reported comorbidity index | 1.52 (0.80-2.89) | 1.62 (0.81-3.26) |
| Highly emetogenic treatment (vs. moderately emetogenic) | 1.03 (0.49-2.19) | 1.23 (0.61-2.49) |
| Guideline-consistent antiemetic care (vs. guideline-inconsistent) | 1.26 (0.61-2.62) | 1.08 (0.55-2.12) |
